# P57. Activation of RIG-I induces immunogenic cell death

**DOI:** 10.1186/2051-1426-2-S2-P31

**Published:** 2014-03-12

**Authors:** S Bek, D Kreppel, M Bscheider, CC Lin, T Haas, H Poeck

**Affiliations:** 1 Klinikum Rechts der Isar der TU-München, Medizinische Klinik, Muenchen, Germany

## Background

The interaction between the immune system and cancer cells has become a focus of recent cancer therapy research. Immunogenic cell death, short ICD, was described as a cell death modality that stimulates an immune response against dead-cell antigens, in particular when they derive from cancer cells. Most malignant cells are poorly immunogenic and fail to elicit an effective antitumour immune response. Certain anti-cancer treatments, however, have been shown to induce ICD and transform cancer cells into potent inducers of an anti-cancer immune response. An important component is the induction of a specific cytotoxic T-cell response driven by DCs that have engulfed and processed tumour antigens. Recently, we have shown that the RIG-I ligand 3pRNA induces tumosr cell death *in vitro* and *in vivo*. Whether and how 3pRNA induced tumour cell death leads to a specific antitumor response is unknown. Here we analyse the immunogenicity of RIG-I induced tumour cell death *in vitro* and *in vivo*.

## Material and methods

We induced tumour cell death by treating ovalbumin expressing B16 melanoma cells with 3pRNA. We co-cultured 3pRNA treated B16-OVA cells with splenic DCs and CFSE-labeled OT-I T-cells to analyse a specific T-cell activation and proliferation. Furthermore, we vaccinated 3pRNA treated B16-OVA cells subcutaneously into C57BL/6 mice to analyse their immunogenic potential *in vivo*. After vaccination, draining lymph node cells are analysed for T-cell activation and IFNg production using flow cytometry.

## Results

We show that 3pRNA treatment leads to increased cytokine expression, upregulation of costimulatory molecules, cross-presentation and induction of cell death in B16-OVA melanoma cells *in vitro*. 3p-RNA treated B16-OVA cells induce proliferation and IFNg production of OT-I T-cells in co-cultures with spleen derived DCs. After subcutaneous injection of 3pRNA killed B16-OVA cells but not live B16-OVA cells into C57BL/6 mice, potent proliferation and IFNg production of antigen specific CD4 and CD8 T cells is seen in the draining lymph node. Overall, these effects were more pronounced after 3pRNA treatment than after Oxaliplatin induced cell death.

## Conclusions

3pRNA treatment of tumor cells leads to a potent immunogenic phenotype with induction of antigen-specific T-cell responses both *in vitro* and *in vivo*. These findings may have implications for a new therapeutic approach in immune mediated cancer treatment.

